# A modular microfluidic bioreactor with improved throughput for evaluation of polarized renal epithelial cells

**DOI:** 10.1063/1.4966986

**Published:** 2016-11-16

**Authors:** Paul Brakeman, Simeng Miao, Jin Cheng, Chao-Zong Lee, Shuvo Roy, William H. Fissell, Nicholas Ferrell

**Affiliations:** 1 Department of Pediatrics, University of California, San Francisco, San Francisco, California 94143, USA; 2 Department of Biomedical Engineering, Vanderbilt University, Nashville, Tennessee 37232, USA; 3 Department of Medicine, Division of Nephrology, Vanderbilt University Medical Center, Nashville, Tennessee 37232, USA; 4 Department of Bioengineering and Therapeutic Sciences, University of California, San Francisco, San Francisco, California 94143, USA

## Abstract

Most current microfluidic cell culture systems are integrated single use devices. This can limit throughput and experimental design options, particularly for epithelial cells, which require significant time in culture to obtain a fully differentiated phenotype. In addition, epithelial cells require a porous growth substrate in order to fully polarize their distinct apical and basolateral membranes. We have developed a modular microfluidic system using commercially available porous culture inserts to evaluate polarized epithelial cells under physiologically relevant fluid flow conditions. The cell-support for the bioreactor is a commercially available microporous membrane that is ready to use in a 6-well format, allowing for cells to be seeded in advance in replicates and evaluated for polarization and barrier function prior to experimentation. The reusable modular system can be easily assembled and disassembled using these mature cells, thus improving experimental throughput and minimizing fabrication requirements. The bioreactor consists of an apical microfluidic flow path and a static basolateral chamber that is easily accessible from the outside of the device. The basolateral chamber acts as a reservoir for transport across the cell layer. We evaluated the effect of initiation of apical shear flow on short-term intracellular signaling and mRNA expression using primary human renal epithelial cells (HRECs). Ten min and 5 h after initiation of apical fluid flow over a stable monolayer of HRECs, cells demonstrated increased phosphorylation of extracellular signal-related kinase and increased expression of interleukin 6 (IL-6) mRNA, respectively. This bioreactor design provides a modular platform with rapid experimental turn-around time to study various epithelial cell functions under physiologically meaningful flow conditions.

## INTRODUCTION

Epithelial cells grow and mature into a polarized monolayer under the correct growth conditions and with an appropriate porous growth substrate, compared to a less differentiated phenotype and a more flattened morphology when grown on non-porous substrates (Fuller and Simons 1986; Steele *et al.*, 1986; Parry *et al.*, 1987; and Orosz *et al.*, 2004). Renal epithelial cell pro-inflammatory responses and cell signaling pathways are commonly studied because renal epithelial cells are often damaged by drugs and ischemic injury that cause inflammation and cytokine release. Normal physiologic function, cell signaling, inflammatory responses, and cell survival all depend on renal epithelial cells having a porous growth substrate and attaining full polarization (Balcarova-Stander *et al.*, 1984). *In vivo*, renal epithelial cells experience tubular fluid flow and apical fluid shear stress that are important in maintaining normal physiological function, and fluid flow is altered under pathologic conditions (Canton *et al.*, 1979; Hostetter *et al.*, 1981; and Hostetter *et al.*, 1981).

It is known that fluid shear stress affects epithelial cell function *in vitro* by altering cytoskeletal architecture (Essig, Terzi *et al.*, 2001), cell-cell junction organization (Duan, Gotoh *et al.*, 2008), apical membrane transporter trafficking (Duan *et al.*, 2010 and Jang *et al.*, 2011), and apical protein uptake (Ferrell *et al.*, 2012 and Raghavan *et al.*, 2014). Primary cilia (Nauli *et al.*, 2003), integrins (Alenghat *et al.*, 2004), and signaling pathways involving the mitogen-activated protein kinase (MAPK) family, which include extracellular signal-regulated kinases (ERK)-1 and 2 (Flores *et al.*, 2011), have all been implicated in the cellular response to fluid shear stress. However, the molecular mechanisms that mediate flow induced mechanotransduction in renal tubular epithelial cells and the implications for disease progression are not fully understood. In order to study renal epithelial cell physiology and pathophysiology under more physiologically relevant conditions requires growing the cells on a porous substrate to full polarity with apical shear flow.

Several microfluidic culture models have been developed specifically for studying aspects of shear dependent cell behavior, transport, and toxicity in renal epithelial cells (Ferrell *et al.*, 2010; Jang and Suh 2010; and Jang *et al.*, 2013). These microfluidic culture systems have incorporated shear flow and a porous substrate for growth but are single use devices and/or are closed systems. Having a closed system can complicate accessing cells for evaluating barrier function and polarization. In these microfluidic systems, cells are seeded and allowed to adhere and differentiate in the device either under static conditions or under constant perfusion. Renal tubular epithelial cells in culture require several days to weeks to achieve complete polarization and a mature epithelial phenotype (Balcarova-Stander *et al.*, 1984 and Wieser *et al.*, 2008). This limits throughput, increases likelihood of device failure, and complicates direct comparison with static controls. A modular system that allows application of well calibrated apical shear stress to cells grown on modular porous substrates that can be rapidly moved into and out of the device would provide several significant advantages for studying epithelial cell physiology including: (1) allowing epithelial cells to reach full polarization and a more-physiological state prior to initiating flow, (2) facilitating rapid retrieval of cells for analysis including mRNA isolation and analysis of unstable phospho-proteins, (3) allowing seeding of cells ahead of time making it possible to monitor monolayer integrity and polarization prior to application of flow, (4) and allowing comparison between identically prepared cells under either static or shear flow culture conditions. To our knowledge, there are currently no reusable modular microfluidic flow systems that allow rapid insertion and retrieval of polarized epithelial cells grown on a porous substrate.

Here, we describe a modular microfluidic system utilizing commercially available porous culture inserts that allows for easy assembly and rapid experimental turn-around. Using this system, we demonstrate that: (1) cells can be routinely monitored for monolayer integrity (inulin leak) and polarization (trans-epithelial electrical resistance—TEER) prior to shear stress application and (2) the ability to rapidly perfuse and retrieve fully differentiated cells is well suited to evaluating short term effects of shear stress on unstable signaling pathways such as protein phosphorylation and cytokine mRNA expression. This device provides a novel platform to study polarized epithelial cells under a variety of temporal and physiologic shear flow conditions while providing a modular system to capture the advantages of pre-experiment monitoring and maturation as well as the ease of assembly for rapid experimental turn around.

## METHODS

### Bioreactor design and fabrication

**FIG. 1.**
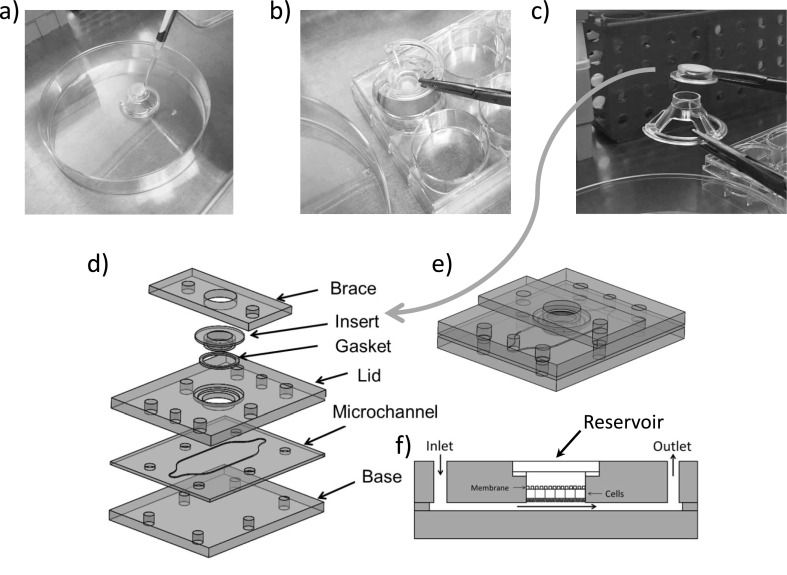
(a) Procedure for seeding cells. Cells are placed on an inverted Snapwell in 200 *μ*l of media and allowed to adhere (b) the Snapwell is inverted into a 6-well plate for further growth. Note the cells are inverted at this point with the apical surface facing down. (c) The cell containing inset is separated from the support structure for insertion into the device. (d) Exploded view of the bioreactor. A PDMS microchannel defines the flow path. The Snapwell inset is secured in the lid of the bioreactor with a brace and sealed with a PDMS gasket. The inlet and outlet ports were tapped with 10–32 threaded luer fittings. (e) Assembled bioreactor (f) Cross-sectional view of the bioreactor (not to scale). The lid is designed such that the Snapwell insert sits flush with the flow path.

**FIG. 2.**
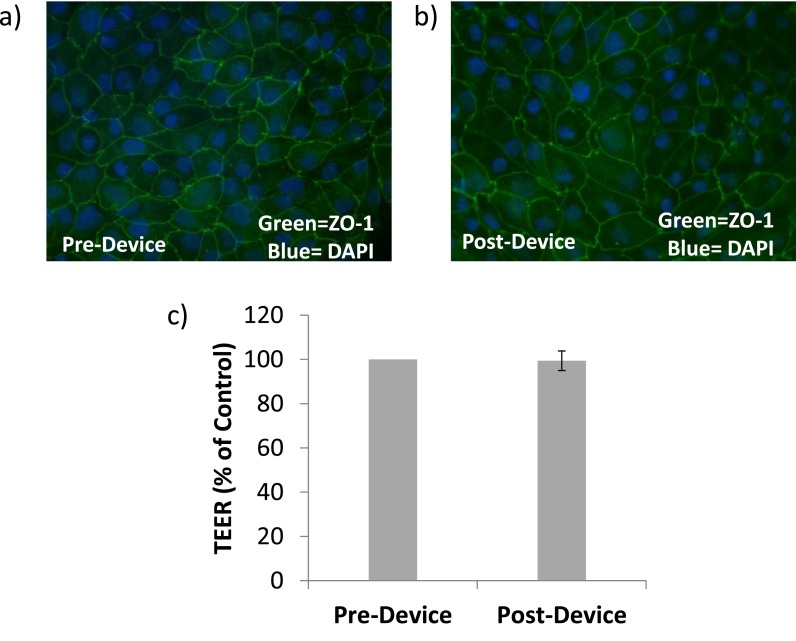
(a) and (b) Representative immunohistochemistry of cells before and after flow indicating the continued health of the renal epithelial cells after being exposed to apical flow. Note stable monolayer phenotype with zona occludins-1 (ZO-1) staining in a cobblestone pattern as expected for renal epithelial cells. Scale bar = 20 μm. (c) Transepithelial resistance (TEER) does not change after assembling the flow chamber prior to initiation of flow. TEER was measured for inserts under static conditions prior to assembly and then after assembly but before initiation of apical shear flow.

The bioreactor was designed to provide perfusion of culture medium across the apical surface of cells grown on the underside of a Snapwell insert (Corning, Snapwell) with an overlying reservoir to allow transport of fluid and solutes across the cell layer. Exploded, assembled, and cross-sectional schematics of the bioreactor system are shown in Figures 1(d)–1(f). A microchannel was created by laser-cutting a flow path out of a sheet of polydimethylsiloxane (PDMS) that is sandwiched between two polycarbonate plates (Figure 1(d), “Microchannel”). The Snapwell insert is a commercial polycarbonate membrane attached to a support structure that allows the insert to be suspended in a standard 6-well culture dish (Figures 1(a)–1(c), “Insert”). These inserts have been widely used for static culture of monolayers of many types of cells. For our experiments, the Snapwell support structure is inverted (Figure 1(a)). Cells are then seeded on the inverted membrane of the insert. The insert is then reverted and placed back into a 6-well culture dish for culturing until use (Figure 1(b)). At the time of insertion into the flow device, the membrane-containing insert is detached from the support structure (Figure 1(c)) and inserted into a cutout machined into the top plate (Figure 1(d)) and a seal is obtained using a PDMS gasket. The top (basal) chamber of the bioreactor consists of a 800 *μ*l reservoir that is easily accessible through a cutout in the brace once the device is assembled (Figure 1(f), Reservoir). Assembly and seeding of cells were performed in a laminar flow tissue culture hood to avoid contamination. We examined the effect of this manipulation of the membrane on the cell barrier function and monolayer integrity. Figure 2 shows that placement of the membrane into the device did not significantly change the morphology of cells nor the TEER of the cells.

**FIG. 3.**
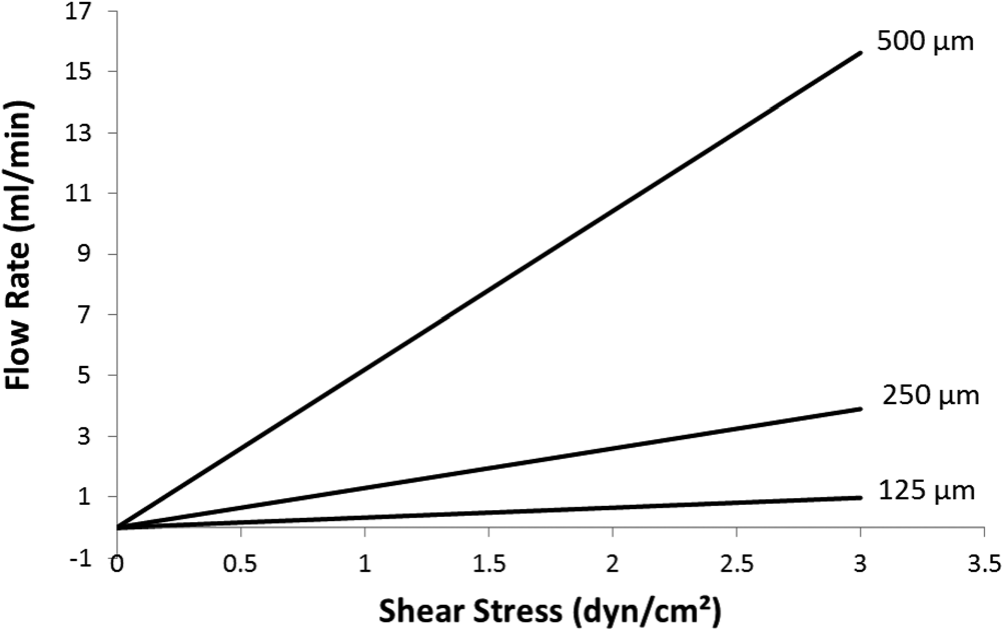
Graphical representation of the shear relationship between flow rate, channel height, and shear stress.

The flow channel underneath the membrane and facing the apical surface of the cells was designed to provide shear stress at the cell surface that approximates *in vivo* conditions. Published data on proximal tubule ultrastructure and flow rates in rats suggest a shear stress in the range of approximately 0.5–5 dyn/cm^2^ under normal conditions (Chou and Marsh, 1987). The height of the laminar flow channel is defined by the thickness of the laser cut PDMS sheet and can vary in height from 125 *μ*m to 1 mm or more. For our experiments, we used channel heights of 125 (data not shown) - 500 *μ*m (Figure 5). The relationship between channel geometry, pump flow rate, and shear stress is shown in Figure 3 and is described further elsewhere (Ferrell *et al.*, 2010). Compression of the gasket was controlled by a step machined into the lid that created consistent height of compression. Physical measurements of the Snapwell inserts demonstrated a variability +/−11 *μ*m (SEM) from the mean height of 3611 *μ*m which translates to a corresponding variability in the channel height. With this variability, we chose to use a 500 *μ*m channel height for our experiments to limit the percent of channel height variability.

### Cell culture and immunohistochemistry

Human renal epithelial cells (HRECs) (Innovative Biotherapies, Inc.) were cultured at 37 °C in 5% CO_2_ atmosphere. Cells were maintained in UltraMDCK medium supplemented with 1 ml/l insulin, transferrin, ethanolamine, and selenium (ITES), 0.7 *μ*g/l triiodothyronine (T3), 50 *μ*g/l epidermal growth factor (EGF), 100 I.U./ml Penicillin, and 100 *μ*g/ml Streptomycin. This medium was used for all subsequent HREC cultures.

Cells were plated on the underside of Snapwell inserts at a density of 5 × 10^5^ cells/cm^2^. The Snapwell inserts were placed in the incubator under static conditions for 4 h to allow for cell attachment. Inserts were then inverted and reattached to the Snapwell support ring and cultured normally. Immunohistochemistry was performed using previously published protocols (Ferrell *et al.*, 2010).

### Transepithelial electrical resistance

Transepithelial resistance was measured with an EVOM2 Ohmmeter (World Precision Instruments). Resistance was measured prior to cell attachment and the blank resistance was subtracted from subsequent measurements. TEER was measured once per day. The resistance measurements were normalized to account for the area of the membrane, resulting in units of Ω-cm^2^.

### Inulin leak

The rate of inulin diffusion across the cell layer was measured daily. FITC-labeled inulin (F3272, Sigma) was dissolved in UltraMDCK medium at 100 *μ*g/ml and 3 ml was introduced to the apical side of the cells. Inulin-free media was added to the basolateral side. The membranes were incubated at 37 °C for 2–24 h depending on how long the cells had been in culture, and the media on the basolateral side of the cells was collected. The concentration of FITC-inulin in the collected medium was measured using a fluorescent plate reader with 475 nm excitation and 500–550 nm emission. The inulin leak rate was calculated by multiplying the concentrations by basal media volume (500 *μ*l) to obtain FITC-inulin mass (*μ*g). This value was divided by the FITC-inulin incubation time and normalized to the membrane surface area to yield the inulin leak rate (*μ*g/cm^2^/day).

### Perfusion

Once confluent monolayers formed on the Snapwell insert, the insert was removed from the ring and placed into the bioreactor. The bioreactor was connected to a media reservoir with an inline bubble trap between the pump head and the bioreactor inlet. Flow of media was controlled by a peristaltic pump. For protein analysis, cells were exposed to 2 dyn/cm^2^ shear stress for 10 and 30 min at 37 °C in 5% CO_2_. For RT-PCR, cells were exposed to the same shear stress for 5 h. Control samples were maintained by the same procedure, but with no exposure to shear stress.

### Western Blots

Cells were collected in lysis buffer, sonicated, and centrifuged for 10 min at 10,000g. Supernatants were collected and protein was quantified using either Bradford or BCA assays. Equal amounts of protein were separated on SDS-PAGE gels and transferred to PVDF membranes. Samples were blocked in 5% milk (total protein) or 5% bovine serum albumin (phosphorylated protein) and probed with rabbit anti-ERK (9102, Cell Signaling) or phospho-ERK (9106, Cell Signaling) antibody overnight at 4 °C. Membranes were washed 3× in Tris-buffered saline with 0.3% Tween 20 (TBST) and incubated in goat anti-rabbit HRP secondary antibody for 1 h at room temperature. Membranes were washed 3× in TBST and developed with the WestFemto Supersignal chemiluminescence substrate.

### RNA isolation and Real-Time PCR

Total RNA was isolated using the Micro RNeasy kit (Qiagen). RNA quality was determined by measuring absorbance at 260 nm and 280 nm on a Nanodrop Spectrometer. First-strand cDNA was synthesized from total RNA primed with oligo(dT) using Superscript III reverse transcriptase (Invitrogen, San Diego, USA) according to the manufacturer's instructions. Real-time PCR was performed on triplicate samples using the Applied Biosystems 7500 Real-Time PCR System for expression of human IL-6 (Applied Biosystems Assay ID # Hs00985639_m1), TIMP1 (Applied Biosystems Assay ID # Hs00171558_m1), TIMP2 (Applied Biosystems Assay ID # Hs00234278_m1), and MMP9 (Applied Biosystems Assay ID # Hs00234579_m1). Data were normalized to human GUS mRNA levels (Applied Biosystems Assay ID # Hs00939627_m1) as an endogenous control. Relative expression (RE) levels are expressed relative to static control using the ΔΔCt formula (RE = 2−[(Ct(gene, test sample) − Ct(GUS, test sample)) − (Ct(gene, static sample) − Ct(GUS, static sample))]), in which CT is the threshold cycle number. ΔΔCt and RQ Manager Software version 2.3 (Applied Biosystems) were used to determine CT numbers.

## RESULTS AND DISCUSSION

**FIG. 4.**
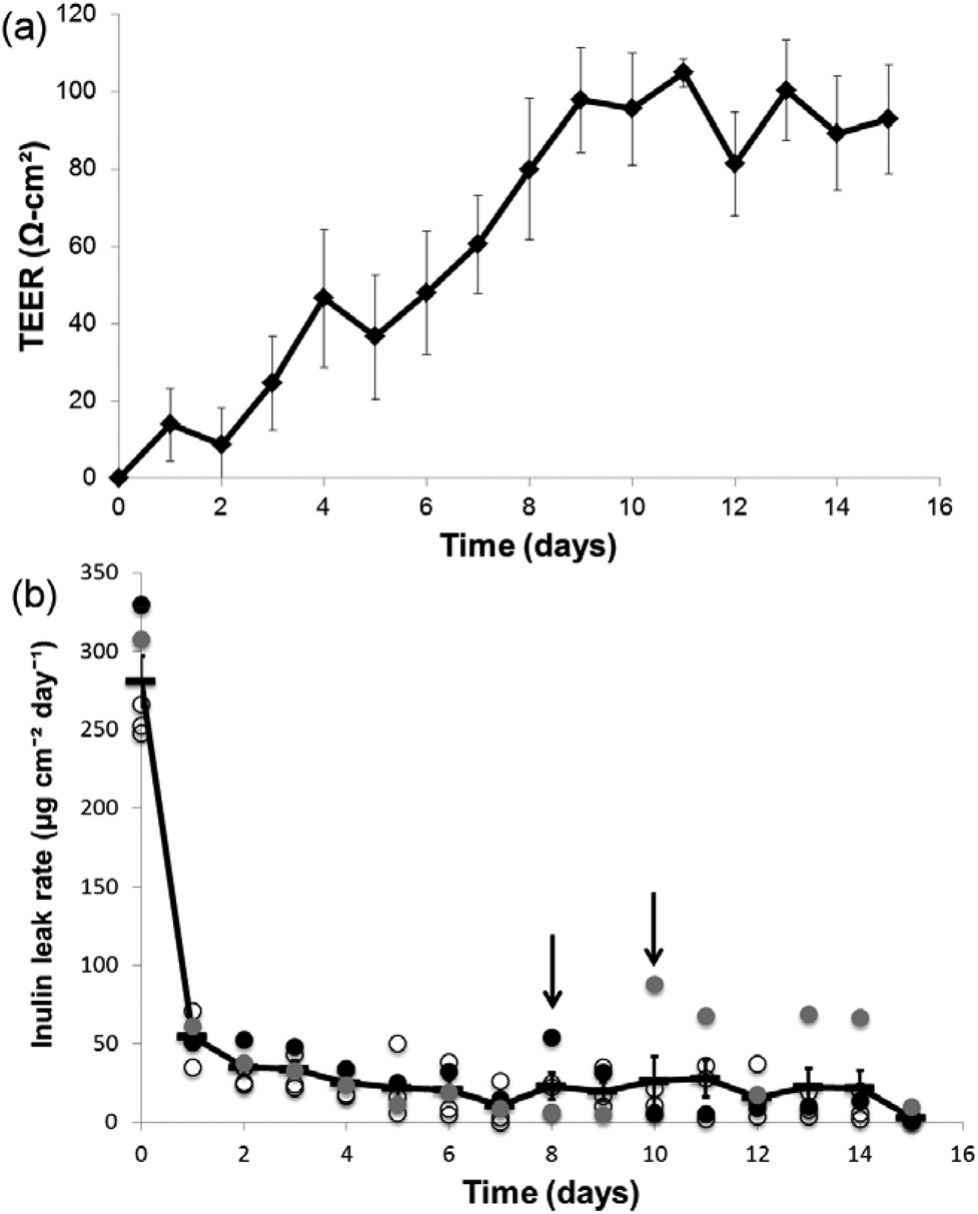
(a) TEER versus time for HREC cells grown on the underside of Snapwell inserts. TEER plateaued around day 9. Data are given as the mean ± standard error (SE) (n = 6) (b) Inulin leak rate of HREC cells. Inulin leak decreased dramatically between days 1 and 2 and steadily decreased thereafter. Minimal inulin leak was detectable in the majority of the samples by day 7. Leaking samples were easily distinguished as indicated by the arrows on the plot.

Figure 4 shows the maturation of the epithelial monolayer integrity over 2 weeks prior to being inserted into the bioreactor device for flow experiments. Transepithelial electrical resistance (TEER) was measured daily to determine the integrity of the cell monolayer and to evaluate the “tightness” of cell-cell junctions. Figure 4(a) shows that the TEER increased steadily after seeding and stabilized at approximately day 10 with a steady state TEER of ∼100 Ω-cm^2^. Renal tubular epithelial cell TEER can vary significantly depending on the origin of the cells. Proximal tubular epithelial cells generally have lower TEER consistent with a looser epithelium while distal tubular epithelial cells have higher TEER corresponding to a tighter epithelium. The steady state resistance of ∼100 Ω-cm^2^ is consistent with other cultured renal tubular epithelial cells of proximal tubule origin (Wieser *et al.*, 2008 and Elwi *et al.*, 2009). To evaluate the barrier function, inulin leak was measured. Inulin is not actively taken up by tubular epithelial cells and thus makes a convenient marker for paracellular leak. Inulin leak rate versus time is shown in Figure 4(b). As expected there is a considerable leak of inulin across the cell layer early after seeding the cells. The leak rate decreased inversely with the increase in TEER as the cells proliferate and junctional complexes mature. At approximately day 10, most of the samples showed minimal inulin leak. However, the data sets colored in black and grey in Figure 4(b) show that two of the samples had a spike in inulin leak as denoted by the arrows. This is likely due to development of a small monolayer defect. The data show that the inulin leak decreased in the days following the spike suggesting that the cells were able to cover the denuded area.

These data show that primary human renal epithelial cells are able to form confluent monolayers with barrier function on commercially available Snapwell membranes. The combination of TEER and inulin leak measurements provided a convenient method to screen for monolayer integrity and barrier function. This assay allows for identification of samples that can be excluded from experiments based on insufficient barrier function and shows that even when the cell monolayers are damaged, they are able to recover from small leaks in the cell layer.

**FIG. 5.**
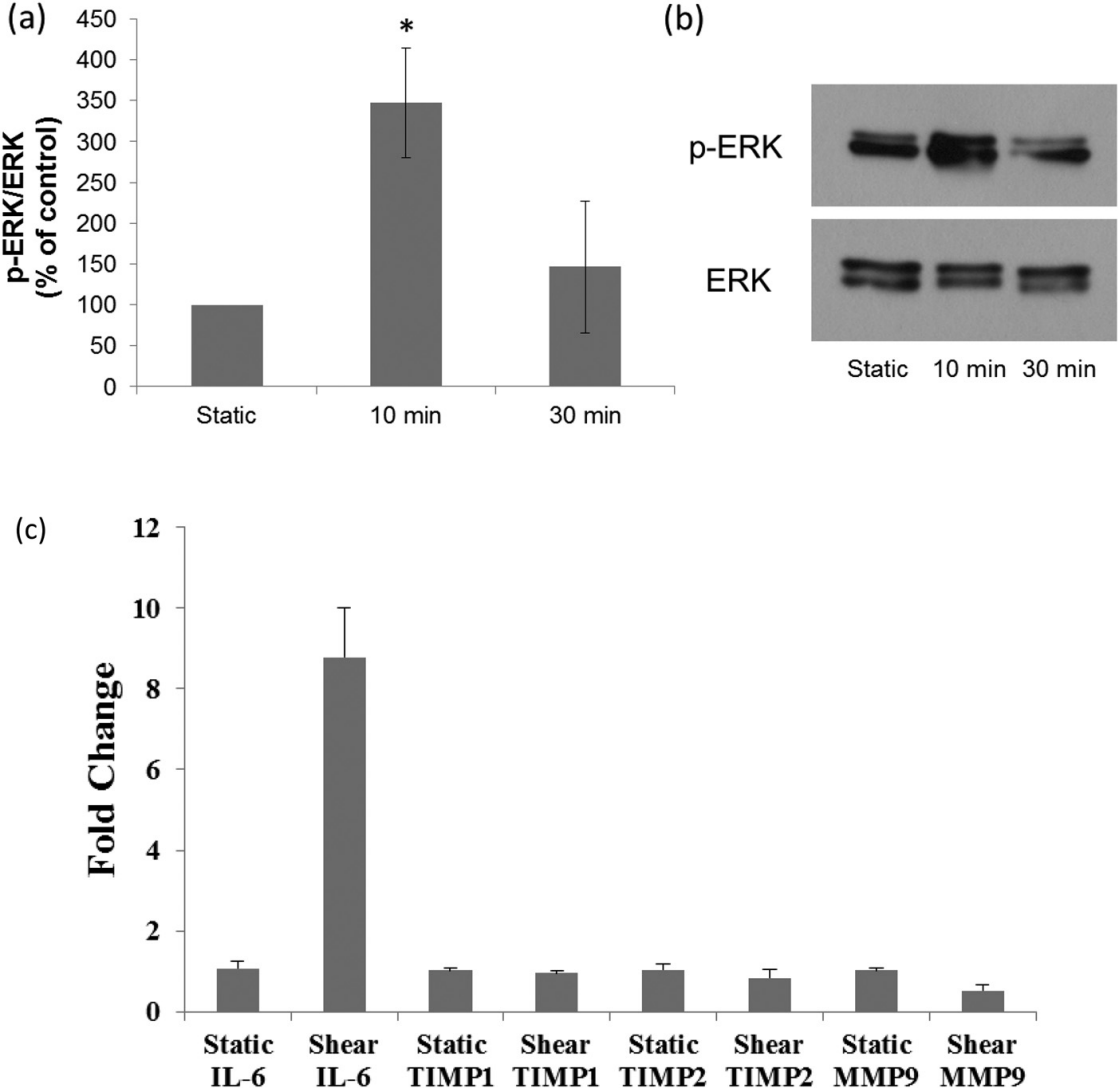
(a) Densitometry measurements of total and phosphorylated ERK under static conditions and after exposure to 2 dyn/cm^2^ shear stress for 10 min and 30 min. (b) Representative western blot of ERK and p-ERK in HREC cells exposed to shear stress for 0, 10, and 30 min (n = 4 for each timepoint). The asterisk signifies the significant difference between p-ERK at 10 min of shear compared to static (p < 0.05) (c) Evaluation of the effect of shear on mRNA expression of IL-6 and protease proteins. Cells were exposed to 2 dyn/cm^2^ shear stress for 5 h or static culture conditions. RNA was harvested using standard methods and qRT-PCR was performed for IL-6, TIMP1, TIMP2, and MMP9. Results are shown as the ΔΔCt ± standard error (SE) (n = 4) and demonstrate a significant increase in IL-6 mRNA following exposure to 5 h of shear (** denotes (p < 0.05)).

Next we used our modular system to evaluate the effect of fluid shear stress on rapid and transient changes in cell signaling. ERK phosphorylation was measured by western blot in HREC cell exposed to 2 dyn/cm^2^ shear stress for 10 and 30 min. Figure 5 shows that ERK is phosphorylated in response to shear stress at 10 min (p < 0.05). By 30 min ERK phosphorylation was not statistically different from baseline. These data illustrate the utility of the rapid retrieval of cells in this system for measuring short term effects of shear stress on cell signaling. ERK phosphorylation has been shown to mediate shear stress induced changes in inflammatory cytokine RNA expression in collecting duct epithelial cells (Flores *et al.*, 2011). In that study, ERK was phosphorylated within 10 min of shear stress application and continued up to 120 min following application of shear stress. In other studies on renal epithelial cells, ERK phosphorylation has been shown to increase and decrease back to baseline in as little as 10 min in response to chemical stimulus (Su *et al.*, 2010). Differences in the time course of ERK phosphorylation between our studies and previous studies on the effect of shear stress may be due to differences in the cells used (primary human versus mouse collecting duct) and the level of shear stress (0.4 dyn/cm^2^ versus 2 dyn/cm^2^).

We also examined the effect of initiation of fluid shear stress on the mRNA expression of inflammatory cytokine IL-6 and proteins involved in matrix remodeling, all of which are important in the response to renal injury. Cytokines and proteins involved in matrix remodeling have all been shown to contribute to the pathophysiology of acute kidney injury (AKI) (Bengatta *et al.*, 2009; Liu *et al.*, 2009; Dennen *et al.*, 2010; Lee *et al.*, 2011; Basile *et al.*, 2012; Levitsky *et al.*, 2014; and Meersch *et al.*, 2014); however, little is known about their regulation by apical shear stress. As shown in Figure 5, at 5 h post-induction of shear flow there is a significant increase in the expression of mRNA for Interleukin-6 (IL-6) but not proteases TIMP metallopeptidase inhibitor 1 (TIMP1), TIMP metallopeptidase inhibitor 2 (TIMP-2) nor matrix metallopeptidase 9 (MMP-9). Of note, with the rapid retrieval of the membrane support from the device, we achieved excellent yields of mRNA. In addition, it is possible to split the membrane support after retrieval from the device and isolate both mRNA and protein from the same experimental condition (data not shown).

This novel, modular bioreactor system provides advantages for a number of applications. For short term and medium term perfusion experiments, where changes in cell signaling, RNA expression, or protein expression induced by fluid flow are of interest, this system provides significant improvements over current closed microfluidic systems because cells can be easily accessed and monitored for differentiation and polarization prior to application of shear stress. Furthermore, direct comparison with static controls is simplified using this system. Systems that use non-porous substrates such as glass coverslips as culture substrates are not appropriate for study of epithelial cell transport or polarity as a porous growth substrate is essential to development of differentiated, polarized, transport competent cells. In summary, we have designed and fabricated a bioreactor system that allows for rapid assembly and disassembly of a microfluidic flow system using mature epithelial cells and can be used for to evaluate the cellular response to application of physiological flow conditions.
